# Archetype symbols and altered consciousness: a study of shamanic rituals in the context of Jungian psychology

**DOI:** 10.3389/fpsyg.2024.1379391

**Published:** 2024-05-09

**Authors:** Hang Sun, Eunyoung Kim

**Affiliations:** Japan Advanced Institute of Science and Technology, Nomi, Ishikawa, Japan

**Keywords:** Jungian psychology, archetype symbols, altered states of consciousness, shamanic rituals, shamanism

## Abstract

The alteration of consciousness during shamanic rituals is both a physical and mystical phenomenon. It involves psychological and spiritual experiences. Through ritual practices, shamans can connect with archetype within the collective unconscious, utilizing trance-inducing techniques for “hallucinatory exploration”. This study surveyed 75 participants to investigate the impact of prototype symbols in Shamanistic rituals on participants’ consciousness states focusing on Jungian psychology’s concept of archetype. The results indicate that archetype symbols in shamanic rituals can significantly influence participants’ conscious state, leading them to experience a conscious dissolution of the self. Furthermore, archetype symbols have different effects at the stages of consciousness change. In particular, during the “Visionary Restructuralization” stage, archetype symbols, such as patterns, masks, totems and music, brought participants’ consciousness to a peak and caused significant changes to it. These findings suggest that the metaphoric function of archetype symbols plays a crucial role in rituals. Archetype symbols connect the individual to the collective unconscious through visual images and symbolic imagery. They prompt the participants to experience emotional resonances that transcend individual experiences and affect their state of consciousness.

## 1 Introduction

Shamanism can be described as a group of techniques for bridging the real and non-real worlds. Practitioners access the “spiritual world” by using these techniques and achieve a spiritually altered experience of consciousness (Krippner, 2000). Ethnography documents the methods of shamanic practitioners in tribes, whereby, they entered altered states of consciousness; shamans could gain strength and knowledge through spirit worship (Eliade, 1964). They use techniques, such as trance and induction, to stimulate hallucinatory and ecstatic states. In this manner, they engage in “hallucinatory exploration.” This process may involve the use of certain objective elements, such as music (e.g., drumming), dance, recitation of certain texts, and incantations (Santarpia et al., 2021). Research suggests that experienced shamanic practitioners, due to their heightened threshold crossing, such as some shamans who perceive themselves as having died and merged with nature, can rapidly induce altered states of consciousness, gaining access to the spiritual realm. However, ordinary individuals without shamanic training seeking ecstatic states may require specific practices. Noll et al. (1985) suggested that about 90% of people could enter altered states of consciousness, while experiments by Rock indicate that even inexperienced participants can achieve altered states through shamanic consciousness-altering techniques (Rock et al., 2013). Additionally, pre-existing unusual beliefs and emotional biases within shamanic rituals are also significant factors influencing the extent and characteristics of such anomalous experiences among general participants (Polito et al., 2010). These findings suggest that experienced shamanic practitioners may have an easier time entering altered states of consciousness, while laypeople may need to employ specific techniques to achieve similar results. When the ecstatic state emerges, the Shaman accesses the spiritual world. Current research suggests that methods that elicit the shamanic altered states of consciousness (ASC) primarily include sleep restriction, meditation, drug effects, and perceptual deprivation. However, the expectancy effect of participants is also considered to be an important factor influencing states of consciousness. The expectancy effect refers to the anticipation of non-volitional responses that can directly influence an individual’s subjective experience (Kirsch, 2000). Several aspects of ASC require in-depth exploration. Therefore, there are still many facets of shamanic consciousness alteration worthy of further investigation. The process of ASC extends beyond being a physiological or mystical phenomenon; it encompasses profound psychological and spiritual experiences.

Archetype in Jungian analytical psychology offer a firm theoretical foundation for comprehending ASC and the associated symbolic imagery in ritual practices. From Jung’s perspective, archetype are ancient patterns and images that originate in the collective unconscious (Jung and Hull, 1968). These archetype reside in the unconscious and serve as common, universal signs and symbols for human beings. They manifest in various forms, including dreams, myths, and genetic inheritance, and carry psychological and spiritual significance (Neher, 1996). This is consistent with shamans entering an ASC to enquire into the spirit world. Therefore, this study aims to utilize the concept of archetype from Jungian psychology to investigate the impact of archetype symbols in shamanic rituals on participants’ states of consciousness. The phenomenon of altered consciousness in shamanic rituals was explained from the perspective of Jungian psychology. This study adds to the multilevel understanding of the state of consciousness in shamanic rituals, and points the way forward for the application of Jungian psychology, both theoretically and practically.

## 2 Literature review

### 2.1 Shamanism and altered states of consciousness

Altered states of consciousness (ASC) play a central role in shamanism. Traditional altered states of consciousness (ASC) are described as qualitative changes in individual psychological functioning that are distinct from normal waking consciousness (Tart, 1972). Within this conceptual framework, shamanism shows correlations with traditional ASC (Winkelman, 2002). Shamans undergo rituals that induce altered states of consciousness (ASC) and transform them into spirit beings, creating a unique shamanic state of consciousness (SSC). Unlike ASCs, SSCs are considered supernatural encounters within their cultural context. While all SSCs are based on ASCs, not all ASCs are SSCs. Shamanic practitioners use a combination of methods such as fasting, dehydration, sleep deprivation, drumming, chanting, and the ingestion of psychoactive substances to induce altered states of consciousness. The resulting trance experiences, which are archetype of shamanic experiences, serve as vital indicators of the participants’ altered states of consciousness during rituals (Van Pool, 2009).

As a pioneer in shamanic studies, Eliade (2020) was the first to promote the “trance” model, defining the shamanic altered consciousness system as a state of ecstasy manifested through interactions in the spirit world. Additionally, Eliade considers the concept of “soul flight” as a sign of shamans entering the ecstatic trance state and journeying to the realm of spirits (Lagana, 2010). This theory has been widely recognized, providing important insights for understanding shamanic states of consciousness. Peters and Price-Williams (1980) attempted to describe cross-cultural factors indicating shamanic trance by studying ethnographic texts in 42 different languages. They suggested that shamanic trance is a specific type of ASC, involving mastery or control over the onset and duration of ASC, post-trance recall, and the ability to communicate with the audience during ASC. However, religious critics have expressed skepticism toward Eliade’s views, arguing that his perspective is overly subjective and fails to account for the cross-cultural nature of religion, lacking objective descriptive analysis of religious phenomena (Allen, 1988).

In contrast, Winkelman (2004) interpreted trance as an experience of “soul flight” that occurs in the spirit as a result of the transformation of the brain’s neural structures. This is an important symbol that distinguishes shamanism from other religions. In addition, Winkelman (2011a) suggested that shamanic conscious experience was a product of the brain function and neural structure. Shamans can achieve changes in consciousness during ritual practices through various hypnotic induction techniques (Winkelman, 1986). This highlights that in shamanic practice, the guidance of the ASC is closely linked to the physiological and neurological mechanisms of the brain. This view finds support in archeology. Evidence from the caves of Lascaux in present-day southern France suggests that shamans may use remote, quiet, and completely dark caves to achieve ultimate sensory deprivation, such as the cat room in Lascaux (Lewis-Williams, 1997). Ethnographic and archeological evidence has been provided for methods of consciousness change in certain areas. For example, the tribal shamans from the Amazon and African regions utilize external agents (e.g., cactus, mandala, and other external media). A trance state occurs through the internal stimulation of drugs, hence, the shaman can rapidly access an altered consciousness during a religious ceremony and achieve a religious experience (Fotiou, 2019). This approach has become commonplace in the induction of ASC worldwide (Schultes et al., 2001). The experience of using drugs to control ASC varies depending on the psychoactive agent and other methods of achieving ASC. Although the physiological consistency and structure of the human nervous system limit how ASC experiences are encountered, such constraints create consistency in some shamanic practices (Lewis-Williams, 1997). This consistency involves interactions between experiences within and outside the participants’ bodies. Humans undergo extreme emotional experiences, including visual and auditory hallucinations, sensations of flying or swimming, and feelings of fear and possible death (Van Pool, 2009). Harner (1973) further demonstrated this approach by explaining that the trance induced by the shaman’s use of drugs during rituals takes the form of “lucid dreaming.” It is worth noting that Harner (1990) successfully simulated shamanic journeys by replicating the objective conditions of the rituals, enabling even inexperienced participants to experience altered states of consciousness.

Although many scholars have shown interest in the concept of “shamanic consciousness state,” it still lacks a clear definition and has not been fully understood, which has led to some controversy (Rock and Krippner, 2007). Some scholars regard the shamanic consciousness state as a subject of study in physiology or mystical phenomena. However, we must recognize that shamanic states of consciousness involve deeper psychological and spiritual experiences that still deserve further exploration today. In this regard, Cardeña and Beard (1996) make an illuminating point. They suggest that the altered consciousness identified in shamanic deep hypnosis is most likely a product of the same innate biological and cognitive tendencies as that of ancient primates. This aligns with the Jungian concept of archetype. Jung considered innate products as archetype and proposed that their psychological influence can impact an individual’s state of consciousness through unconscious images (Jung, 2014). This provides a fresh perspective for explaining the phenomenon of consciousness change from a psychological standpoint. This comprehensive study is expected to deepen the understanding of the phenomenon of consciousness change and offer valuable insights for future research in the field of psychology.

### 2.2 Jung and shamanic practice archetype

In Jungian psychology, the archetype plays a vital role. According to Jung (2014), archetype are innate, spontaneously generated psychological patterns, symbols, or images that exist in the collective unconscious of the brain and are not affected by external influences or transmissions. They are believed to be hard-wired patterns in the subconscious mind and are found in mythology, religion, art, and literature from different cultures. Shamanism, as a religious culture, provides important examples of Jung’s theory of archetype (Merchant, 2006; Sandner and Wong, 2013; Scott, 2014). In shamanism, archetypes are understood as inner patterns latent in the individual psyche, manifested in rituals, myths, and traditional practices. Shamanic archetype involve the natural world, the spiritual realm, and other elements. They are fundamental symbols in individual and collective consciousness, expressed and embodied through rituals and myths (Peters and Price-Williams, 1980).

There have been numerous discussions about the intersection of Jungian psychology and shamanism (Haule, 2010). Jung formalized the concept of the psyche as the source of spiritual or “supernatural” experiences in his concept of the collective unconscious, archetype, and processes of individuation (Scott, 2014). Jung posited that the collective unconscious encompasses universal and innate forms within humanity, generating tendencies or archetype (Jung and Hull, 1968). Within this theoretical framework, individuals shape their psychological structures through the process of individuation, by engaging with and internalizing the archetype within the collective unconscious. Similarly, Shamanism also emphasizes seeking direct experiences of the inner world through imagined inner beings such as spirit helpers or archetypal figures, considering these inner entities to be real existences (Commisso, 2011). These experiences are considered direct perceptions of the spiritual realm, echoing the interaction between the individual and the collective unconscious in Jungian psychology. Overall, Jung and shamans have the study of the soul and soul loss in common. Both are instances of the same primitive image or instinctive pattern known to the world (Bright, 2009). Whether it’s the hallucinatory experiences during shamanic rituals or the symbolic imagery of Jungian psychological archetype, images play a crucial role.

Jung identified the psychic as images. He interpreted the mental images present in the collective unconscious state as archetype characteristics of the individual (Pietikainen, 1998). He suggests that these archetype are more than just symbols or references to known external factors; they are images of an emerging unconscious content struggling to find a way to express itself (Jung and Hull, 1968). These archetype symbols exist in the mental world of the individual in the form of an unconscious state (archetype images usually take the form of humans or animals or a fusion of the two) and are triggered through special scenes and fixed patterns (James Hollis, 2002). These archetypal symbols exist within the individual’s spiritual realm as manifestations of the unconscious, triggered by specific scenarios and fixed patterns. Caputo’s empirical research, using eye and mirror gaze, provides compelling evidence in support of this notion. Specifically, by placing mirrors in dimly lit rooms, Caputo et al. (2021) demonstrates how easily dissociative experiences, potentially involving archetypal imagery, can be induced in “normal individuals.” These experiences include depersonalization, derealization, and identity separation. Lange et al. (2022) describes dissociative experiences as the process of “derealization, depersonalization, and identity separation” within individuals. This idea of ‘progressive dissociation’ may refer to the sequential firing or ‘layered’ involvement of brain regions. From the initial early processing of the external world (such as visual facial features), gradually moving to the intermediate stage of processing the internal world (such as the sense of ownership of the body and face), and finally involving the processing of identity and self, thus realizing the archetypal symbolic triggering process in the unconscious state. In contrast, the dissociative experiences induced by shamanic archetypal symbols require the induction of an unconscious state through ritual to be perceived, that is, enter the spiritual world to achieve a change in consciousness (Winkelman, 2002). Although shamans may believe that the entities they encounter in the spirit world exist independently (Winkelman, 1990), Jung’s understanding of these experiences is based on his model of the psyche (Bright, 2009). Jung explained the shaman’s spiritual world as an expression of the collective unconscious and an archetype transcending humanity (Scott, 2014). Noll, who was influenced by Jungian psychology, saw the shaman’s spiritual world as a manifestation of the inner psychological imagery. This reinforced Jung’s concept of archetype emerging from the collective unconscious (Noll et al., 1985).

Current research shows that the interpretation of shamanic archetype favors a theory of innateness derived from a biological basis (Merchant, 2016). Jung argued that archetype are innate, a view that crystallized in the context of shamanism (Merchant, 2012). In shamanism, archetype include elements of characterization, song, and dance that form the “consensus hallucination” of the community (van Löben Sels, 2019). This “consensus hallucination” is closely linked to the spiritual practices of shamanism (Sandner and Wong, 2013). In esthetic and therapeutic rituals, the “*a priori*” concrete expression of these archetype can be observed (Herrmann, 2015). For example, drums are used by shamans and their people to induce exodus, enter a state of spirituality, and reconnect with the energies of others and the universe (Eliade, 2020). Another aspect of the explanation stems from emergentist, which holds that specific circumstances and personal experiences can be important sources of shamanic archetype (Merchant, 2012). This theory, which appears to have originated with Pietikainen (1998), regards archetype as “symbolic forms” rather than biologically based. Roesler (2012) pointed to culture and socialization as central to the experience of the archetype. The archetype is embellished or enriched by materials from the environment and the individual’s experience. Therefore, they usually take the form of humans or animals, or a fusion of the two, such as totems and natural elements of earth, water, fire, and wind (Scott, 2014). Based on these two interpretations of shamanic archetype, Knox (2004) proposed the emergence/development model. He saw archetype as an emergent phenomenon, the result of neurobiological structures formed through the developmental experiences of an infant’s early life. It is the result of interactions between the human organism and its environment during development (Merchant, 2006). Knox focused her discussion on the early mind/brain development of the human infant’s perceptual processes and employed Johnson’s pictorial model of the image as an early mental structure, thus, meeting the need for a model that provides both the archetype and the archetype image (Johnson, 1989; Merchant, 2006). This model is supported by different scholars and has been verified in Siberian shamanism.

Overall, although the current research on shamanic archetype and altered states of consciousness has sparked significant interest among scholars, for example, Winkelman (2011b) explored the cross-cultural origins, nature and social transformations of shamans and other mystical religious healers. He also utilized neuropsychology to explain altered consciousness phenomena and proposed psychological models. However, research to explain altered states of consciousness from a psychological perspective is still relatively limited. Although Noll et al. (1985) acknowledges the central role of vivid mental imagery in intentionally induced altered states of consciousness, he does not provide detailed explanations of the origin or nature of these images. He only suggests that they seem to arise spontaneously from the individual’s psyche. This suggests that psychology still has relatively limited explanations for altered states of consciousness phenomena. Therefore, this paper seeks to comprehensively elucidate the impact mechanisms of archetype symbols on altered states of consciousness during the ritual process by integrating various sources of shamanic archetype, such as patterns, masks, animal totems, and natural elements.

## 3 Methods

### 3.1 Participants

This study was conducted from August to November 2022. Researchers recruited participants from the northeastern region of China to take part in shamanic rituals. Participants were not authentic shamans but individuals who either believed in shamanism or were interested in it. There were no age, education, or gender restrictions. All the participants were voluntarily and independently recruited by the researcher. People under the age of 18 were excluded from this survey. Based on the above criteria, a total of 75 eligible participants accepted the questionnaire, including 41 males (M.age = 35.02, range = 25–51, SD = 6.62) and 34 females (M.age = 34.59, range = 24–48, SD = 6.78). We provided participants with a comprehensive informed consent form, which explained the purpose and methods of the research. In addition, participants were informed that their data would be anonymized, collected, processed, and storage of their data.

### 3.2 Materials and procedure

The shamanic ritual practices involved in this study were conducted in a generally accepted setting. We provided participants with a comprehensive informed consent form, which explained the purpose and methods of the research. In addition, participants were informed that their data would be anonymized, collected, processed, and storage of their data. Before the commencement of the ritual, participants were required to view prototype symbol images commonly present in daily shamanic, such as masks, patterns, totems, and other symbolic representations. Researchers provided explanations about the symbolic significance of these archetypal symbols to the participants.

To understand the overall tendency of participants’ beliefs during the rituals, researchers used the Revised Paranormal Belief Scale (RPBS) to analyze the participants’ characteristics before the rituals. The scale was originally designed by Tobacyk et al. (1988) and officially released in 2004. However, Tobacyk (2004) discussion ignores gender and age response biases, so Lange et al. (2000) revised Paranormal Belief Scale remains a more accurate choice. The scale revised by Lange is more reliable in terms of reliability. It includes seven dimensions – traditional religious beliefs (e.g., the soul continues to exist even though the body may die), Psi (e.g., mind reading is not possible), witchcraft (e.g., there are actual cases of witchcraft), superstition (e.g., black cats can bring bad luck), spiritualism (e.g., reincarnation does occur), extraordinary life forms (e.g., life exists on other planets), and precognition (e.g., physics can be used for accurately predicting the future). Lange divided the scale into two dimensions: “New Age Philosophy” (11 items, Rasch reliability = 0.90) and “Traditional Supernatural Beliefs” (5 items, Rasch reliability = 0.74). The average scores for both dimensions of the Rasch-RPBS were 25 (SD = 5), and multiple studies have confirmed their construct validity.

During the ritual practice, participants are asked to close their eyes and are accompanied by an 8-beat monotonous drumbeat. During the ceremony, the organizer needs to guide the participants to first imagine that the archetypal symbolic images explained earlier are emerging before their eyes. Imagining these archetypal symbols helped participants embark on a shamanic journey, experiencing a sensation of ascent associated with the archetypal symbols. The organizer will then use guiding words to lead participants through established shamanic journey ritual training. After the conclusion of the ritual, participants are required to rate the effects of archetype before and after the ritual practice and complete the Aussergewohnliche Psychische Zustande (APZ) questionnaire. This was developed by Dittrich (1998) to measure the intensity of ASC. It has been translated into several languages and is recognized worldwide. The APZ includes three stages of ASC – Oceanic Boundlessness (OSE), Fear of Ego Dissolution (AIA), and Visionary Restructuralization (VUS). The OSE has 13 items, the AIA has 22 items, and the VUS has 14 items. The APZ serves as a systematic way to measure and categorize changes in ASC during shamanic rituals, and its validity has been confirmed in various fields. The APZ questionnaire provides a deeper understanding of participants’ changes in the ASC before and after the ritual practice.

Furthermore, participants were still required to complete the Ego-Dissolution Inventory (EDI) questionnaire. The EDI (Nour et al., 2016) is an eight-item questionnaire to assess peak experiences of ASC. Sample items include statements like “I experienced a dissolution of my ‘self’ or ‘ego’.” Participants could sequentially respond to these statements, ranging from “No, no more than usual” (1) to “Yes, I experienced this completely” (100). The internal consistency of the inventory was found to be very good. Ego dissolution is a key feature in rituals for determining whether ASC occurs. The EDI is specifically designed to measure this experience and gain a better understanding of the psychological phenomena that may occur during rituals.

### 3.3 Data analysis

Quantitative analysis was conducted using IBM SPSS Statistics, version 27, Release 27.0.0.0, 64-bit version. Descriptive analysis was initially performed to report the sample characteristics of the survey participants, which included presenting the mean and standard deviation of the sample. To assess the internal consistency of participants’ belief tendencies before the ritual, a Paranormal Belief Survey was utilized. The results were then analyzed using Cronbach’s alpha to determine the internal reliability coefficient, which yielded a reliability coefficient of 0.62 (the generally accepted standard is greater than 0.6).

Researchers conducted a non-parametric binomial analysis on the three subscales of APZ. The null hypothesis (H0) posited that the consciousness alterations identified by EDI are not linked to the altered states of consciousness (ASC) at various stages. By observing the changes in *p*-values, one can decide to reject the null hypothesis (significance indicated as *p* < 0.05). To assess the effectiveness of prototype symbols in inducing and influencing individual ASC experiences, researchers compared the average changes between the datasets before and after the ritual. Using paired sample t-tests, the researchers observed changes in p-values to determine the level of significance (with a general guideline of <0.01), assessing how prototype symbols influenced individual ASC experiences.

Additionally, they constructed a correlation matrix between “ego-dissolution,” “alterations in consciousness,” and “prototype symbols” by calculating Pearson correlation coefficients when p-values were less than 0.05 or 0.01. This analysis aimed to examine the overall ASC awareness induced by prototype symbols. Finally, by establishing three sets of linear regression models, they evaluated how various prototype symbols influenced different ASCs occurring during shamanic rituals. Statistical significance was determined by summarizing the changes in p-values reflected by various prototype symbols in the model (when *p* ≤ 0.05), thus assessing their impact on altering consciousness processes.

## 4 Results

### 4.1 The effect of archetype symbols on participants’ state of consciousness

To address the first objective, that is, to assess the role of archetype symbols on ASC experiences during shamanic rituals, the sample characteristics were reported and a one-sample *t*-test was performed on the APZ and EDI scales to see whether they differed from each other.

Table 1 presents data from the Paranormal Belief Scale, described using mean and standard deviation. In the New Age Philosophy cluster, the average score is 26.185 (SD = 0.759), suggesting participants have high belief levels influenced by interpersonal relationships and external events, with little variability. In contrast, the Traditional Paranormal Beliefs cluster has an average score of 26.570 (SD = 1.085), indicating relatively high belief levels influenced by cultural traditions, with greater variability. Average scores for both clusters exceed the scale’s mean of 25, indicating a generally high level of Paranormal belief before the ritual, which aligns with the study’s criteria.

**Table 1:** Description analysis of the revised paranormal beliefs scale.

	Mean	Std. Deviation	Min	Max
Traditional	3.333	0.509	2.000	4.500
PSI	3.057	0.637	1.750	4.750
Witchcraft	2.863	0.662	1.500	4.000
Superstition	3.093	0.594	1.333	4.333
Spiritualism	3.197	0.652	1.750	4.500
Extraordinary	2.916	0.680	1.333	4.333
Precognition	2.980	0.685	1.500	4.500
**Purified Clusters**				
Cluster 1: New Age Philosophy	26.185	0.759	24.32	27.97
Cluster 2: Traditional Paranormal Beliefs	26.570	1.085	24.81	29.02

To investigate the impact of archetype symbols on the ASC experience during rituals, we conducted a non-parametric analysis (Table 2). We converted the value obtained from survey data into binominal data to categorize the variables into two groups of “High” and “Low.” Medium value is adopted to classify the “High” and “Low” group. Binomial analysis results indicate the following conclusions:

**Table 2:** EDI and ASC binomial test analysis of various stages.

Items		Category	*N*	Observed Prop.	Test Prop.	Exact Sig (2-tailed)
OSE	Group1	2	42	0.56	0.50	0.356
	Group2	1	33	0.44		
	Total		75	1.00		
AIA	Group1	2	47	0.63	0.50	0.037*
	Group2	1	28	0.37		
	Total		75	1.00		
VUS	Group1	2	47	0.63	0.50	0.037*
	Group2	1	28	0.37		
	Total		75	1.00		
EDI	Group1	2	13	0.17	0.50	0.000**
	Group2	1	62	0.83		
	Total		75	1.00		

*p < 0.05, Category 2 = “High,” Category 1 = “Low”.

Null Hypothesis (*H0*): There is no association between consciousness alterations detected by the Ego-Dissolution Inventory (EDI) and different stages of altered states of consciousness (ASC).

At the OSE stage, we fail to reject the null hypothesis (*p* = 0.356, *p* < 0.05), suggesting that the consciousness alterations detected by EDI may not be related to this stage. This indicates that the observed data at the OSE stage are consistent with the testing hypothesis. At the AIA and VUS stages, we reject the null hypothesis (AIA: *p* = 0.037, VUS: *p* = 0.037, *p* < 0.05), indicating a significant difference between the observed data and the testing hypothesis. It suggests that the consciousness alterations detected by EDI may be associated with these stages.

### 4.2 Overview of ASC awareness induced by archetype symbols

The second aim was to conduct paired samples t-tests on the mean changes in ASC before and after the ritual. Meanwhile, the correlation matrix of the EDI, ASC stages, and archetype symbols is constructed to provide an overview of the ASC induced by archetype symbols.

Table 3 shows the mean values of participants’ attitudes toward changes in states of consciousness before and after the religious ceremony. The results indicate an upward trend in the mean level of consciousness at the end of the religious ceremony. Using paired samples t-tests, significant differences were observed in the effectiveness of different archetype symbols in guiding changes in consciousness during the rituals. Specifically, masks (*t*(75) = 3.968, *p* < 0.01), natural elements (*t*(75) = 3.665, *p* < 0.01), animal totems (*t*(75) = 5.551, *p* < 0.01), shamanic dances (*t*(75) = 7.742, *p* < 0.01), and shamanic music (*t*(75) = 4.879, *p* < 0.01) showed significant increases. However, patterns (*t*(75) = 2.192, *p* = 0.032) showed a slightly lower level of significance, despite a slight upward trend.

**Table 3:** Ritual: archetype symbols & ASC stages survey.

Measure	Mean		Standard deviation	
	Before	After	Before	After
Pattern	2.65	2.86	0.76	0.82
Masks	2.35	3.01	0.79	1.05
Natural Elements	1.97	2.27	0.72	0.91
Animal Totems	2.52	3.08	0.84	0.96
Shamanic Dance	2.49	3.42	0.72	0.82
Shamanic Music	2.51	3.13	0.86	1.02
APZ: altered states questionnaire				
Oceanic Boundlessness (OSE)	2.20	3.64	0.71	0.88
Dread of Ego Dissolution (AIA)	3.59	3.77	1.41	1.11
Visionary Restructuralization (VUS)	3.11	3.95	0.95	0.93

In addition, the paired-sample *t*-test results of the ASC experience before and after the religious ceremony showed that during the shamanic religious ceremony, participants exhibited a significant increase in religious experiences in the Ocean Unbounded stage (*t*(75) = 10.922, *p* < 0.01) and the Visionary Restructuralization stage (*t*(75) = 7.082, *p* < 0.01). However, this trend was not significant during the Dread of Ego Dissolution stage (*t*(75) = 0.918, *p* = 0.362). These findings indicate significant differences in the impact of archetype symbols on participants at different stages of the ASC experience, particularly in the Dread of Ego Dissolution stage, where their interfering effect is relatively small.

Tables 4, 5 demonstrate the use of the Pearson correlation coefficient to assess the relationship between EDI (ego-dissolution) and other variables. Specifically, based on the correlation matrix, EDI shows significant correlations with VUS, patterns, masks, animal totems, and shamanic music. The results indicate that during the VUS, influenced by the aforementioned four types of archetype symbols, participants’ ASC reached a peak, leading to a conscious experience of ego-dissolution.

**Table 4:** Correlation of EDI (ego-dissolution) with effects on changes in consciousness.

	EDI	OSE	AIA	VUS
EDI	1			
OSE	−0.011	1		
AIA	−0.044	0.192	1	
VUS	0.255*	0.241*	0.421**	1

*p < 0.05, **p < 0.01.

**Table 5:** Correlation between EDI (ego-dissolution) and archetype symbols in ritual practice.

	EDI	Pattern	Masks	Natural Element	Animal Totems	Shaman Dance	Shaman Music
EDI	1						
Pattern	0.272*	1					
Masks	0.249*	−0.154	1				
Natural Element	0.041	−0.150	0.082	1			
Animal Totems	0.252*	−0.072	−0.042	0.147	1		
Shaman Dance	0.056	0.164	0.087	0.063	0.145	1	
Shaman Music	0.259*	0.070	−0.103	0.108	0.114	0.028	1

*p < 0.05, **p < 0.01.

### 4.3 Correlation factors between archetype symbols and changes in consciousness

The third objective was to determine the influence of archetype symbols in religious practices on different stages of consciousness change. To accomplish this, the study performed a regression analysis on various archetype symbols observed in shamanic rituals.

Table 6 depicts a linear regression model, reflecting the relationship between Models 1, 2, and 3 and the religious art in the three ASC stages. The data obtained from the three sets of models showed that the F-test results for Models 1 (*F* = 2.973, *p* = 0.012 < 0.05), 2 (*F* = 3.671, *p* = 0.003 < 0.05), and 3 (*F* = 4.217, *p* = 0.001 < 0.05) were all meaningful. The VIF values in all three models are less than 5; hence, there is no covariance problem.

**Table 6:** Linear regression models of archetype symbols and stages of consciousness change.

Independent variable	Model 1		Model 2		Model 3	
	Coefficients	95% CI	Coefficients	95% CI	Coefficients	95% CI
Pattern	0.330** (2.746)	0.095 ~ 0.566	0.174 (1.174)	−1.016 ~ 2.441	0.308* (2.527)	−0.232 ~ 0.139
Masks	0.207* (2.214)	0.024 ~ 0.390	−0.053 (−0.464)	−0.116 ~ 0.464	−0.046 (−0.491)	0.027 ~ 0.456
Natural Elements	0.151 (1.399)	−0.061 ~ 0.364	−0.020 (−0.148)	−0.279 ~ 0.172	0.242* (2.205)	−0.237 ~ 0.170
Animal Totems	−0.035 (−0.340)	−0.236 ~ 0.166	0.202 (1.602)	−0.281 ~ 0.242	−0.033 (−0.318)	−0.110 ~ 0.363
Shamanic Dance	0.074 (0.620)	−0.160 ~ 0.308	0.315* (2.146)	−0.045 ~ 0.450	0.126 (1.046)	0.100 ~ 0.477
Shamanic Music	−0.184 (−1.933)	−0.370 ~ 0.003	0.339** (2.895)	0.027 ~ 0.604	0.288** (2.993)	−0.232 ~ 0.139

Model 1 = Oceanic Boundlessness (OSE); Model 2 = Dread of Ego Dissolution (AIA); Model 3 = Visionary Restructuralization (VUS); *p < 0.05; **p < 0.01.

Specifically, in the OSE model, two prototypical symbols that significantly influenced the change in the ASC intensity were identified. The archetype “pattern” showed a regression coefficient of 0.330 (*t* = 2.746, *p* = 0.008 < 0.01), while “masks” showed a regression coefficient of 0.207 (*t* = 2.214, *p* = 0.030 < 0.05). In particular, “pattern” had a more substantial effect on the intensity of the initial stage of consciousness change, as evidenced by a significant increase in the intensity of the first stage of consciousness change with the use of this archetype symbol in ritual practice. In the AIA model, the regression coefficients were 0.315 (*t* = 2.146, *p* = 0.035 < 0.05) for shamanic dance and 0.339 (*t* = 2.895, *p* = 0.005 < 0.01) for shamanic music. This indicates that the presence of both elements, shaman music and shaman dance, contributed to an increase in the intensity of participants’ ASC during the Dread of Ego Dissolution stage. In particular, shaman music had a significant effect on the prevalence of ASC during the AIA stage. In the VUS model, the regression coefficients for “pattern” were 0.308 (*t* = 2.527, *p* = 0.014 < 0.05), “natural elements” were 0.242 (*t* = 2.205, *p* = 0.031 < 0.05), and “shamanic music” were 0.288 (*t* = 2.993, *p* = 0.004 < 0.01). All three results showed a significant correlation. This suggests that the archetype symbols of painting, papercut, and shamanic music led to a significant increase in the intensity of consciousness alteration during the ritual practice in the third stage of Visionary Restructuralization, enabling the participants to enter ASC more quickly.

## 5 Discussion

This study investigated the impact of archetype symbols in rituals on the induction and shaping of ASC experiences. The EDI and APZ revealed that the participants experienced significant changes in consciousness following the ceremony.

### 5.1 Impact of archetype symbols on ASC experience

The study aimed to investigate the impact of archetype symbols on the ASC experience during rituals. The results of the binomial test analysis demonstrate significant changes in participants’ states of consciousness during the AIA and VUS stages of altered states of consciousness (ASC). The participants experienced a conscious ego-dissolution.

To investigate the extent of archetype symbols’ influence on consciousness stages, the study conducted paired-sample *t*-tests on the average changes in consciousness before and after shamanic rituals (Table 3). The study’s outcomes indicated an upward trend in the mean values of Oceanic Boundlessness, Dread of Ego Dissolution, and Visionary Reconstruction post-ceremony. The most significant change was observed in the mean value of Oceanic Boundlessness. This outcome can be ascribed to three factors. First, the specificity of the ritual environment exerted a significant influence on these changes. According to Jung (2014), archetype symbols are adorned or enriched by materials drawn from the environment and the individual’s experiences. Therefore, the uniqueness and context of the ritual environment may play a key role in enhancing the impact of archetype symbols on the states of consciousness (Stevens, 2021). This perspective is supported by Caputo’s empirical research on “gazing into eyes and mirrors,” which suggests that dim lighting and the refraction of mirrors can induce experiences involving archetypal imagery. These experiences may lead to phenomena such as personality depersonalization, derealization, and dissociated identity (Caputo et al., 2021). Lange et al. (2022) proposed a model of “progressive dissociation” involving reality derealization, depersonalization, and dissociative identity. This concept of gradual dissociation may involve the continuous triggering or hierarchical engagement of brain regions. This process facilitates the activation of archetype symbols in unconscious states. Similarly, the shamanic process of triggering archetype also necessitates a secluded external environment. Shamans construct a unique threshold space by leveraging common factors among participants. This may encompass specific brain structures, mythological legends, genetics, and other shared elements within the participant population (Lewis-Williams and Clottes, 1998; Winkelman, 2002). For example, shamanic rituals typically take place at night. Through large-scale gatherings held at night, participants can more easily access the unconscious spiritual realm, which facilitates alterations in consciousness (Eliade, 2020) Consequently, the ritual environment plays a pivotal role in altering consciousness by engendering specific psychological processes that enhance participants’ receptivity to archetype symbols, ultimately leading to corresponding changes in consciousness.

Secondly, researchers believe that participants’ pre-existing belief level was an important factor in this result (Polito et al., 2010). Higher levels of paranormal beliefs may have increased sensory changes during the rituals, which would have a more significant effect on ASC (Vaitl et al., 2005). The results of the survey on paranormal beliefs indicate that all surveyed participants exceeded the mean level. Such psychological expectancy effects provide the necessary prerequisites for participants to enter altered states of consciousness more rapidly (Bandura, 1978). Compared to the other two stages, we observed a noticeable upward trend in OSE. This trend is closely associated with participants’ visual perceptions. Williams’ research suggests that participants in the OSE stage may experience geometric visual concepts, including dots, grids, and other winding lines within geometric shapes (Kluver, 1926; Lewis-Williams and Dowson, 1990). These concepts are projected onto real visual perceptions and partially obscured. Since the same neural systems in participants enable everyone to potentially experience internal images, participants’ states of consciousness show a significant improvement. The average for the Dread of Ego Dissolution phase surpassed the baseline level. This contradicts previous findings where scholars asserted that the encounter with Dread of Ego Dissolution was subjectively negative and fear-inducing. Such an experience is typically characterized as perilous and undesirable (Hermle et al., 1993).

### 5.2 Profiles of ASC induced by archetype symbols

The second aim of this study was to construct a profile of the ASC induced by archetype symbols. The results of the paired sample t-tests before and after the ceremony (Table 3) indicated the importance of masks, animal totems, and shamanic dance and music as archetype in the ceremony for the ASC experience. The result adds to the evidence that archetype symbols can be consciously perceived when they contain content, especially when they contain recurring symbols and images (Jung, 2014; Scott, 2014). It must be noted that shamanic dance, as an archetype, showed a significant upward trend in mean scores compared to pre-ceremony (Kirmayer, 1993). This finding suggests that archetype symbols represent pre-formed ideas or images, and more deeply represent the meaning given by the body. As the ceremony progresses, the shaman can convey spiritual images through specific dance movements and postures, which affect the participant’s spiritual world. The metaphorical function of archetype and symbols plays an important role in this process. This finding has been consistently reported in various studies. In addition, it is speculated that the emergence of these results is related to the body’s control of the sympathetic nervous system. Shamans employ particular movements and rhythms to trigger a distinctive bodily state in the participants, akin to the effects of sleep deprivation and fasting (Winkelman, 2002). This altered physical state may contribute to an elevated state of consciousness, providing a new dimension for future studies on ASC.

To further explore the impact of archetype symbols on consciousness at different stages of ASC, the study constructed a correlation matrix, including EDI and the three stages of ASC (OSE, AIA, and VUS) with archetype symbols (Table 4). The results indicate that during the VUS, the participants reached a peak in ASC and underwent ego-dissolution. This offers a distinctive insight into the psychological experience of ritual practice. Notably, painting patterns, costume masks, totems, and shamanic music played a significant role in intensifying consciousness changes during the process of visionary reconstruction and emerged as influential factors inducing ego-dissolution. The results are consistent with the phenomenological analysis of rituals. Phenomenological scholars have successfully simulated shamanic rituals through the rhythm of music, constructed a model suitable for experiments, and effectively guided the participants into an exploratory journey within the spiritual world (Harner, 1990). This is consistent with the significant role of archetype symbols, especially shamanic music, in visionary reorganization in this study. This result demonstrates the possible use of archetype symbols in guiding changes in consciousness. In shamanic rituals, the symbolic imagery of archetype symbols, such as patterns, masks, and totems, is utilized by amplifying the power of shamanic music archetype to prompt the participants to enter a state of collective unconsciousness. This drives significant changes in states of consciousness (Kirmayer, 1993).

In conclusion, archetype symbols played a pivotal role in shaping the ASC profile during the ceremony. This phenomenon can be attributed to the metaphorical functions of archetype symbols, reaching a peak in consciousness intensity during the VUS, accompanied by a moderate ego-dissolution experience. Notably, archetype symbols, such as patterns, masks, animal totems, and shamanic dance and music, guided participants through significant shifts in consciousness. The study posits that these changes may be closely linked to factors, such as the ritual environment’s specificity, sample characteristics, and the regulation of sympathetic nerves during the ritual.

### 5.3 Impact of archetype symbols on ASC stages

The third study aimed to determine the relationship between the different stages of consciousness alteration and the prototypical symbols. To this end, a linear regression model was constructed between the two (Table 5). The results of the model confirm that shamanism and Jungian psychology are manifestations of the same universally recognized primal images or instinctual patterns (Bright, 2009). In addition, the archetype symbols, significant in the model, coincide with the distribution of ritual processes. This may suggest that certain archetype and symbols in rituals are crucial in directing and shaping participants’ states of consciousness.

During the Oceanic Boundlessness phase, the archetype of patterns and masks showed relevance. The study suggests that images serve as a direct pathway to the unconscious (Hillman, 1982). Energy-filled images are considered sacred, and capable of evoking emotional resonances related to the psyche and the self (Jung, 2014). Through the ritual, participants’ mental archetype manifest as symbolic images, achieving image redirection (Brown and Mitchell, 1986). This directs individual perception and attention, facilitating the participants to enter deeper unconscious states (Lyon, 2020). In the Oceanic Boundlessness phase, the materialization of archetype can enhance the psychological effects, making them crucial mediators of the psychological experiences within the ritual.

In contrast, the Dread of Ego Dissolution phase shows prominence for the shamanic archetype of music and dance. This could be due to two reasons. First, it may be connected to shamanic ritual procedures. Shamanic ceremonies are carried out at night. It produces a trance state through social gatherings in dim light (Winkelman, 1986; Eliade, 2020). Shamanic music and dance continue through the process, acting as essential tools for guiding the participants into a state of collective unconsciousness (Winkelman, 2021). Second, it is intimately tied to participants’ emotional components. Previous studies indicate that experiencing distress coincides with the self-dissociative phase of anxiety, resulting in a negative effect (Hermle et al., 1993). At this point, negative emotions have the potential to significantly impair a person’s capacity for processing social stimuli, autobiographical memory, attention, and perception (Phelps, 2006). Within this point, archetype symbols, such as dance and music, may play a significant role in mediating emotions by assisting the participants in navigating difficult emotional circumstances.

During the VUS phase, the consciousness reaches its peak, accompanied by ego-dissolution. Archetype symbols, such as patterns, natural elements, and shamanic music, exhibit statistical significance. This phenomenon can be interpreted through two potential perspectives. Firstly, it may be linked to the perceptual aspects of shamanic rituals. The VUS stage is described by Lewis-Williams and Dowson (1990) as a trance stage, where individuals enter a tunnel through a vortex (Speck, 1976). Visual experiences of animals, humans, and objects are often distorted, eventually leading to a sense of merging with geometric and iconic images. Participants may undergo deep psychological processes related to reality disintegration, depersonalization, and identity dissociation, achieving experiences of self-separation from the body (Lange et al., 2022). Secondly, it may be related to the ritual guidance process. During the VUS stage, participants embark on their final journey guided by drumbeats and guided words. The significance of prototype symbols may reflect their role as triggers in the ritual process (Rock and Krippner, 2007).

### 5.4 Further limitations and future directions

Although there is sufficient data to support the study’s claims, there are certain limitations to consider. The participants in this survey are not genuine shamans but rather individuals who either practice shamanism or have an interest in it. The differing thresholds between these two groups may introduce biases. Due to privacy concerns, researchers did not collect extensive participant characteristics, such as conducting tests for mental illnesses before the ritual, which may limit the generalizability of the study’s findings. The investigation of archetype in this study is based on fundamental research on the universality of shamanism. However, since shamanism is a worldwide primal religion, the archetype in the rituals may vary due to cultural differences. Future research should expand the investigation to include cross-cultural archetype to accurately reflect the diversity of consciousness transformations. Additionally, in the Results section, researchers made adjustments by applying the Bonferroni correction (*α* = 0.05) to the correlation matrix to account for statistically significant results. Unfortunately, the results did not show significant correlations, which may indicate a limitation in our study due to the restricted amount of data collected in a specific setting. Future studies could utilize more advanced data analysis methods, such as multiple regression, to thoroughly investigate correlations. Researchers believe that future studies should incorporate control group methods to provide a more comprehensive understanding of the impact of archetypal symbols on shamanic consciousness transformation experiences. The study sample was selected from areas where shamanism is practiced; hence, it is limited in diversity, which may restrict the ability to generalize the results.

## 6 Conclusion

This study explains the changes to the state of consciousness in shamanic rituals from a psychological perspective based on the archetype concepts of Jungian psychology. The results suggest that multifaceted psychological mechanisms trigger ASC in shamanic rituals. Archetype symbols used in shamanic rituals significantly affected participants’ state of consciousness, causing them to undergo a conscious experience of ego-dissolution that was significantly different from the waking state. The changes in consciousness before and after the ritual showed a significant upward trend, especially in the Oceanic Boundless. Furthermore, through archetype symbols with metaphorical features, the participants experienced significant changes in their consciousness in the VUS. This mechanism suggests that archetype symbols are capable of guiding consciousness into a deeper state, thereby, inducing significant shifts in consciousness during the ritual. In addition, different types of archetype symbols had different effects on the participants at different stages of ASC, highlighting their specific roles in rituals for different stages of consciousness. These findings provide useful insights for a deeper understanding of the mechanisms of consciousness alteration in shamanic rituals and have strong implications for future research.

## Data availability statement

The datasets presented in this study can be found in online repositories. The names of the repository/repositories and accession number(s) can be found at: https://doi.org/10.6084/m9.figshare.24899118.v1.

## Ethics statement

The requirement of ethical approval was waived by the Japan Advanced Institute of Science and Technology Life Science Committee for studies involving humans because it was conducted in a commonly accepted rituals setting. The studies were conducted in accordance with the local legislation and institutional requirements. The participants provided their written informed consent to participate in this study.

## Author contributions

HS: Writing – original draft. EK: Writing – review & editing.
